# Synchronous lymph node involvement by metastatic carcinoma and lymphoma

**DOI:** 10.1002/jha2.419

**Published:** 2022-03-24

**Authors:** Lindsay Bigham, Faisal Rawas, Ramya Vinnakota, Rohit Venkatesan, Ranjana Nawgiri, Jayati Mallick, Kirill A. Lyapichev

**Affiliations:** ^1^ Department of Pathology The University of Texas Medical Branch Galveston Texas USA; ^2^ Department of Internal Medicine The University of Texas Medical Branch Galveston Texas USA

1

A 74‐year‐old female was referred for evaluation of axillary lymphadenopathy found on routine screening mammography. Suspicion for lymphoma involvement was raised. The consequent lymph node biopsy showed synchronous involvement by small lymphocytic lymphoma (SLL) and poorly differentiated metastatic carcinoma of unknown origin. Lymph node flow cytometry was consistent with lymphoma involvement. Core needle biopsy (Figure 1, top left) shows lymphoid tissue heavily infiltrated by large, pleomorphic cells with prominent nucleoli in a background of small monomorphous lymphocytes with irregular nuclear borders concerning for lymphoma. The carcinoma component was positive for CK903 (Figure 1, top center) and p40 and negative for ER, PR, and GATA3, where lymphoma component was positive for CD23 (Figure 1, top right), CD5 (Figure 1, bottom left), and CD20 (Figure 1, bottom center) and negative for SOX11 and CyclinD1 (Figure 1, bottom right). Interestingly, the carcinoma shows CyclinD1 expression. The coexistence of metastatic carcinoma and SLL in a lymph node is a rare event, which is generally associated with poor prognosis.

**FIGURE 1:**
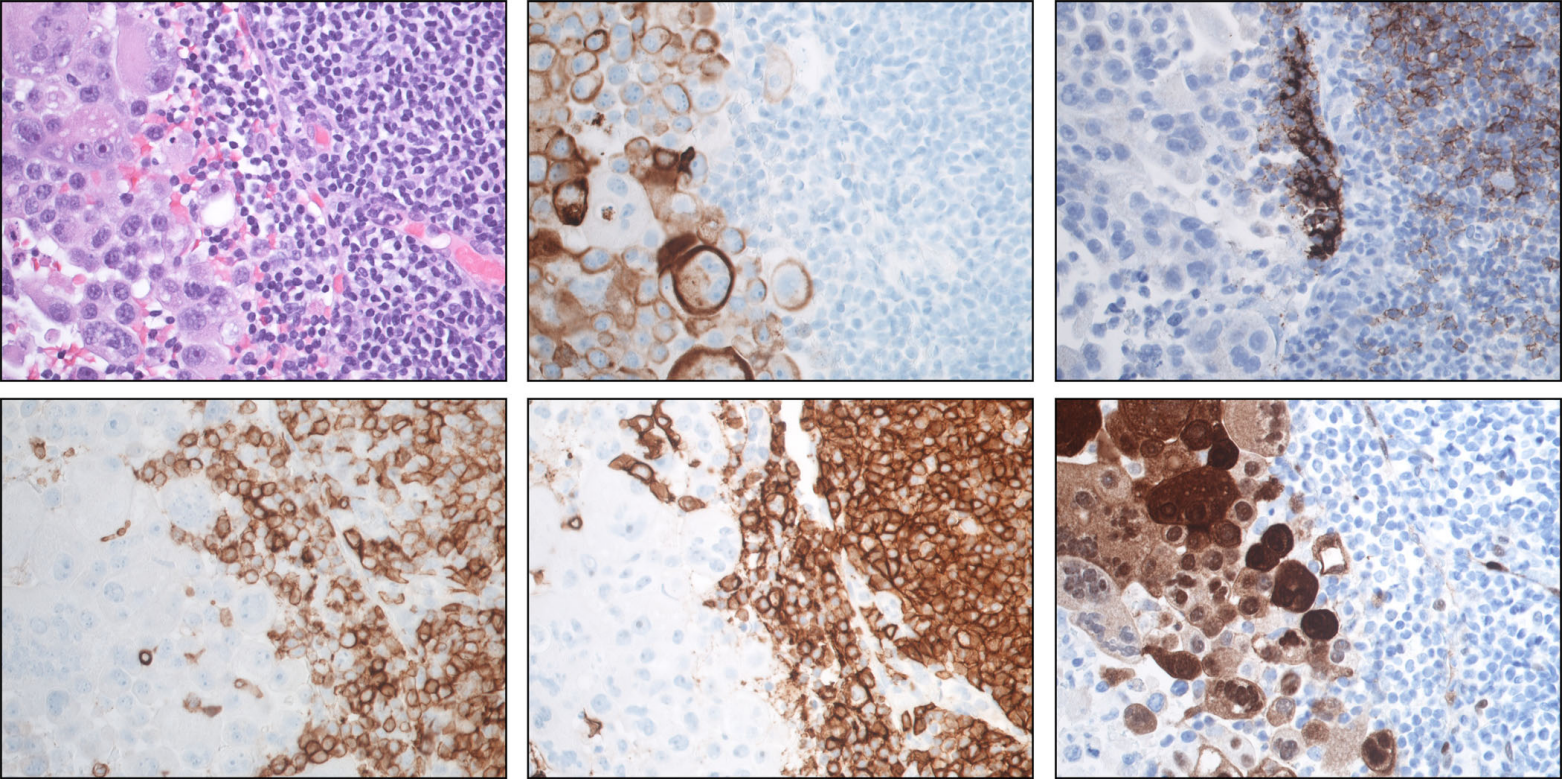
Core needle biopsy shows lymphoid tissue heavily infiltrated by large, pleomorphic cells with prominent nucleoli in a background of small monomorphous lymphocytes with irregular nuclear borders concerning for lymphoma (top left, H&E). The carcinoma component is positive for CK903 (top center), where lymphoma component is positive for CD23 (top right), CD5 (bottom left), and CD20 (bottom center) and negative for CyclinD1 (bottom right). Interestingly that carcinoma cells show CyclinD1 positivity (bottom right)

## FUNDING

None to declare.

## CONFLICT OF INTEREST

The authors declare no conflict of interest.

